# Investigation towards nanomechanical sensor array for real-time detection of complex gases

**DOI:** 10.1038/s41378-025-00899-2

**Published:** 2025-03-24

**Authors:** Md. Abdul Momin, Masaya Toda, Zhuqing Wang, Mai Yamazaki, Krzysztof Moorthi, Yasuaki Kawaguchi, Takahito Ono

**Affiliations:** 1 Graduate School of Engineering, Tohoku University, 6-6-01 Aramaki-Aza-Aoba, Aoba-ku, Sendai, 980-8579 Japan; 2 Department of Bioengineering, University of Pittsburgh, 4200 Fifth Ave, Pittsburgh, PA 15260 USA; 3 R&D Center, Mitsui Chemicals, Inc., 580-32 Nagaura, Sodegaura, Chiba, 299-0265 Japan

**Keywords:** Electrical and electronic engineering, Nanoscience and technology

## Abstract

This study presents the development and characterization of a nanomechanical gas sensor array with piezoresistive detectors for a wide range of applications. The sensors, made of silicon and polymers and integrated with the piezoresistive sensors on a silicon-on-insulator wafer, convert to electrical signals the stress caused by volume change of polymer induced by gas absorption. The fabrication of the sensors incorporates a process where Polymer A (Polyolefin), Polymer B (Fluorocarbon polymer) Polymer C (Acrylic resin), and Polymer D (Amino polymer), are deposited within silicon slits, demonstrating their distinct responses to various vapor species. These sensors show swift response times and efficient recovery periods, which makes them promising for real-time multiple gas and smell monitoring applications. An array of four nanomechanical sensors with polymers shows high repeatability and sensitivity when subjected to multiple gas exposure and turn-off cycles. The gas sensor arrays, effectively monitoring fish quality over several days, suggest a potential for determining optimal storage and early spoilage detection in perishables. The study demonstrates that the nanomechanical sensor array can accurately distinguish between different gas concentrations using principal component analysis, paving the way for real-time, automated multiple gas detection and analysis without human intervention.

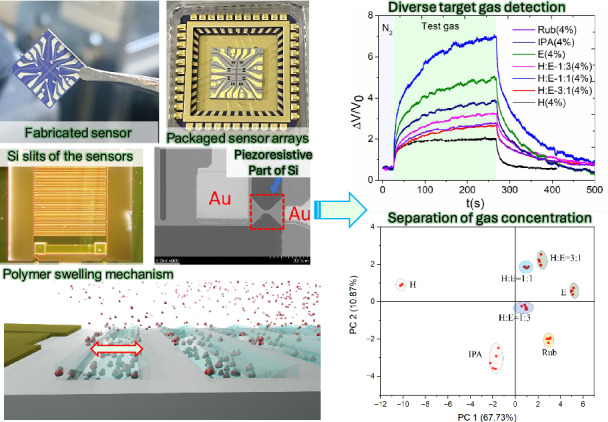

## Introduction

The increasing growth of technology and the different kinds of products we use daily produce different polluting materials, which have become an alarming environmental issue. The polluting species can be toxic, such as carbon monoxide, H_2_S, organic volatiles such as ethanol, acetone, IPA, flammable such as methane, butane, hydrogen, etc. There is a known relationship between disease and exhaled breath constituents. Long-time inhalation of ~ 300 ppm of NO^1^, ~ 1 ppm of acetone^2^, and ~ 2 ppm of NH_3_ can cause asthma, lung cancer, and hepatitis, respectively^3^. The spoilage of various products releases distinct gases and molecules, necessitating their detection for early prediction of product deterioration. These emphasize the need for a groundbreaking gas sensor capable of monitoring environmental conditions, analyzing human breath, and predicting spoilage^4,5^.

To update our environmental air quality, different types of gas sensors have been produced already, for which sensing technology faces new challenges like miniatured size, increased selectivity, low power consumption, sensing diverse species of gases, etc^6,7^. Depending on demands, gas sensors are now operating in different mechanisms like chemoresistance^8^, metal oxide^9^, piezoelectric^10^, piezoresistive^11^, membrane types^12^, nanomechanical gas sensors^13^, cantilever deflection, etc ^12^.

Micro/nanofabrication is a methodology that allows multiple components to be integrated onto a chip in order to miniaturize the sensor systems. Recently, different research groups have developed nanomechanical sensing systems^14,15^. Microfabricated gas sensors, including chemiresistive gas sensors, and field effect transistor-type gas sensors, showed good performance in detecting the gases due to their miniature size, low power consumption, with high reliability^16–18^. Nanomechanical gas sensors detect gas molecules by measuring nanoscale mechanical dimensional changes in response to their presence^9,12–14^. Therefore, the sensitivity, selectivity, and target gas diversity depend on the design of the sensor, choosing the sensing materials along with the deposition technique of the materials. The deposition techniques of the sensing materials used so far are dip-coating, spin-coating, in-situ growth, etc^19,20^. These methods have many issues, such as uncovered area, too much deposition, ununiform deposition alone with highly expensive^19,20^. Most gas sensors like metal oxide, chemosensitive, use different kinds of sensing materials that react with the specific target gases, which reduces the selectivity of the detection of these sensors^21,22^. Conventional sensors, employing various structures and sensing mechanisms, experience drawbacks such as diminished sensitivity, reduced reproducibility, and shortened lifespan. These limitations arise from the chemical reactions involving the sensing materials employed in these sensors^16,23–25^. To address the challenges associated with gas sensing, this study introduces a slit-based nanomechanical gas sensor with a multi-detection feature fabricated through a meticulous microfabrication process^26^. This sensor shows the highest performance in sensitivity among nanomechanical sensors because the stress generated by the buried polymer is concentrated and converted into strain in the piezoresistive sensing element. However, their gas detection sensitivity and detection of gas mixtures had not yet been studied.

This report presents the basic properties and applications of a suspended nanomechanical multisensory array with unique features on a single chip. The sensitivity, reproducibility, and response time of this sensor, which utilizes four functional polymers, are investigated. The applicability of the sensor to gas mixture recognition and food freshness monitoring is investigated.

## Design and working principles of the sensor

**Fig. 1:**
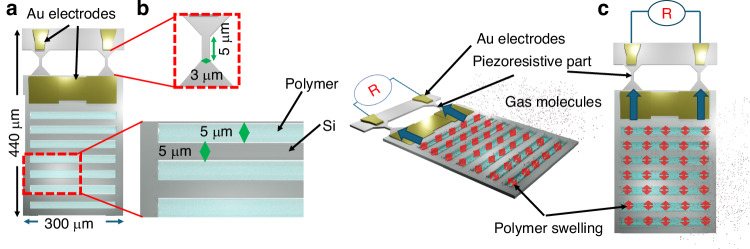
Schematic representation and sensing principle of the sensor. **a** Schematic view of the sensor (top), (**b**) a magnified detail of the silicon slits, and an expanded perspective of the integral piezoresistive sensor part. **c** The sensing principle: gas species diffuse into the polymer deposited into the silicon slits and swell the polymers

Figure 1 (a) shows the top view of the nanomechanical sensor, comprising a piezoresistive sensor and a freely-suspended polymer-filled silicon slits, and Fig. 1b shows the magnified view of the slits part. Gas molecules adsorb onto the polymer, diffuse into the polymer, and change the volume through interaction with polymer molecules^19^. The suspended structure of the polymer slits and sensor parts is designed to concentrate the stresses caused by the volume change of the polymer on the piezoresistive sensor. The nanomechanical multisensory array has been microfabricated using a batch-fabrication process^19^. The multi-sensors recognize a gas mixture from the response pattern of the sensor array. The structural sensitivity of the piezoresistive part and silicon slits have been optimized by simulation using finite element analysis using COMSOL Multiphysics in our previous study^19^. From this study, the size of the piezoresistive sensor part has been selected as 5 × 3 × 7 µm (length× width× thickness). The widths of the Si slits and its opening parts are 5 µm^19^. The width of the Si slits has been selected depending on the sensitivity and the stability after polymer deposition. This is to prevent the slits from stacking due to the volume change of the polymer when the polymer solution is inserted into the slit and dried during the manufacturing process. The length and width of the single sensor are 440 and 300 µm, respectively.

Structurally diverse polymers may serve as selective gas molecule absorbers, inducing volume changes, typically swelling, upon diffusion, with variations in diffusion rates and volume changes among different gas molecules^20^. The sensor converts the physicochemical information from gas adsorption and absorption into mechanical strain, inducing stress in piezoresistive sensors and generating electrical signals. Different gas molecules generate distinct internal stresses, enabling varied resistance changes and diverse gas detection using the same sensor. Operating this stress-detecting gas sensor requires recognizing the in-plane expansion constraint of the swelling polymer by the silicon structure. The surrounding structural rigidity must exceed that of the piezoresistor for effective detection. Comparative sensitivity analysis introduces electro-structural sensitivity ${S}_{\varepsilon }$ ^20^,

1 $${S}_{\varepsilon }=\frac{\Delta R/R}{\varepsilon }$$

where Δ*R*/*R* represents relative resistance change, and *ε* represents the linear driving expansion ratio of the polymer. A simulation illustrates stress and displacement distribution, highlighting the significant stress on piezoresistive components and notable displacement in silicon slits, as shown in Figure S1 (Supplementary material 1).

## Sensor preparation and polymer selection

The nanomechanical gas sensors were fabricated by microfabrication using an SOI substrate (Si/SiO_2_/Si, 7/1/400 µm thickness). Details are described in ref.^19^, also shown in Figure. S2 (Supplementary material 1). Briefly, the process involved boron ion implantation, annealing, SiO_2_ deposition, electrode patterning, and the creation of the piezoresistive part and Si slits using inductively coupled plasma reactive ion etching. Under an optical microscope, four distinct polymers—(i) Polymer A (Polyolefin), (ii) Polymer B (fluorocarbon polymer), (iii) Polymer C (Acrylic resin), and (iv) Polymer D (Amino polymer) are precisely deposited on suspended slits using a needle dispenser (L2015, NTN Corporation) depicted in Figure S2 (A1-A4) (Supplementary material 1). Two cycles of 13 × 8 droplets each were successfully deposited onto the Si slits. Following polymer deposition, resistances increased due to Si slit deformation. However, heat treatments (2 min at a maximum of 180 °C) led to decrease resistances as the slits returned to parallel alignment. Supplementary material 1 Figure. S3 provides a detailed illustration of these changes and the uniformity of polymer deposition, and The Supplementary Material 2 depicts the sensor during polymer deposition using a needle dispenser.

Selected four polymers have the following characteristics. (i) Polymer A is hydrophobic, and water solubility in Polymer A is low, which results in fast saturation of water sorption profiles in such polymers^27^. (ii) The solubility of water and most organic molecules in Polymer B is also relatively lower due to its partially fluorinated backbone^28^. However, Polymer B backbone is polar, and its response can be selective to certain classes of polar molecules. (iii) Polymer C is generally more susceptible to molecule absorption than Polymer A and Polymer B, especially with molecules that can interact with the polar ester groups in its structure. Water absorption is relatively low and well characterized^29,30^ Some alcohols may cause more significant swelling due to better solubility and interaction with the polymer matrix^31^. (iv) Polymer D is a highly polar polymer due to its dense amine group content, making it more reactive towards water and polar molecules like alcohols. It can absorb significant amounts of water, leading to noticeable swelling or volume change^32^. The extent of swelling with molecules would depend on the polarity and molecular size, with smaller, more polar molecules possibly causing more significant volume changes.

**Fig. 2:**
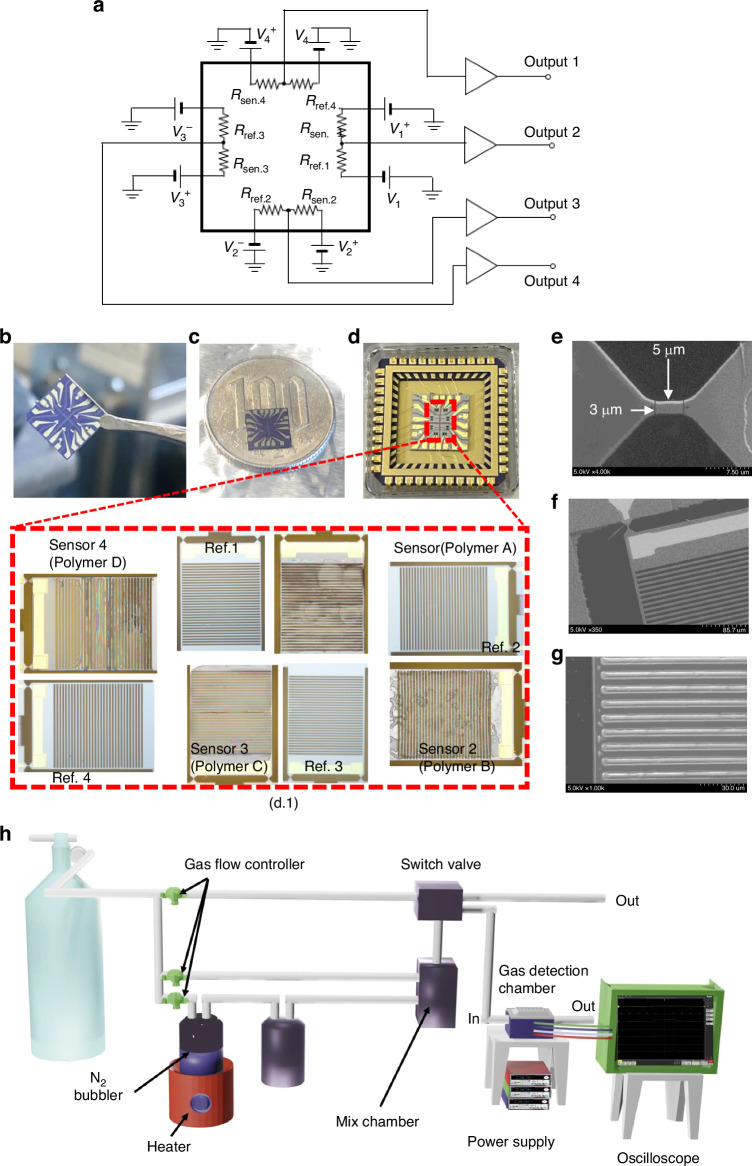
Sensor circuit design, fabrication, and evaluation setup. **a** The circuit design for evaluating the sensors through a gas flow meter. **b**, **c** miniatured sensor arrays where 4 sensors and 4 reference resistors are formed in the same chip. The sensor chip is compared with a 100-yen coin. **d** A fully packaged sensor with deposited polymers and wire bonding [West Bond (MODEL 7700 C)], the images of the sensors after polymer deposition are also shown in (d.1). **e**, **f** and (**g**) show the SEM images of the piezoresistive Si part, Au electrodes and the Si slits, respectively. **h** shows the experimental setup for evaluating the gas sensor using a gas flow meter

The sensor, attached to a 44-pin ceramic case (C-QFJ, Kyocera, Japan) with wax, is electrically connected using an Au wire connector, as depicted in Fig. 2a. The resistance bridges ${R}_{{\rm{sen}}.{\rm{i}}}$ and ${R}_{{\rm{ref}}.{\rm{i}}}$ (i = 1, 2, 3, 4) are balanced using ${V}_{{\rm{i}}}^{+}$ and ${V}_{{\rm{i}}}^{-}$, which are approximately +/–1 V. The balanced center voltages with near zero voltages are amplified a hundred times to the outputs i. Figure 2(b–g) illustrates the miniaturization potential of sensor arrays, which integrate four sensors and four reference resistors on a single chip, roughly the size of a 100-yen coin. These arrays, along with fully packaged sensors showcasing polymer deposited and wire-bonded designs, imply the significant role of polymers in boosting sensor sensitivity, selectivity, and stability.

### Experimental setup for evaluation of the sensors

The experimental setup, evaluation gas chamber, and circuit are displayed in Fig. 2, providing an overview of the testing apparatus. The characterization experiment utilized a small rectangular chamber with the details of the chamber provided in Supplementary material 1 Figure. S4. The output of the sensors was connected to an oscilloscope, and the signals were recorded. The measurements were performed at an ambient temperature of 26–27 ^o^C.

To evaluate the response of the sensors to various partial pressures of analyte, a switching gas flow system was used^20^, as illustrated in Fig. 2h. The system featured two switchable gas flows, dry N_2_ and mixed test gas, generated by combining the flow of saturated analyte vapor with the dry N_2_ flow. The lines for both gases were switched between the sample chamber and the exhaust line through an electrically controlled switch valve. The test gas flow and dry N_2_ flow were carefully controlled using three mass flow controllers. A heater was used outside the bubbler chamber to heat the liquid at 30 ^o^C above the measurement temperature so that the highest saturated gas could be obtained from the gas flow meter.

### Calculation methods

#### Estimation of diffusion coefficients

The dynamics of incorporation of the gas molecules into polymers are often approximated by Fick’s theory of diffusion and absorption^20,33,34^, but non-fickian diffusion in glassy polymers is common^35^. In Fick’s first law, diffusion flux *J* is expressed as *J*$=-{D\; d}\varphi /{dx}$, where $D$ represents the diffusion coefficient, and $d\varphi /{dx}$ is the concentration gradient in depth *x*. This diffusion flux simply represents the movement of adsorbed gas molecules through the polymers of the sensor. The diffusion coefficient of water molecules in polymers, for example, varies significantly depending on the type and properties of polymers. It is not a universal constant and can range from 10^−7^ to 10^−11^ cm²/s for many common polymers^36^. The specific value varies depending on factors such as the molecular structure of the polymer, its porosity, temperature, and the presence of additives or fillers.

Fick’s first law primarily describes static diffusion under steady-state conditions. However, since our sensor response occurs under non-steady state conditions, it is imperative to consider Fick’s second law

2 $$\frac{d\varphi }{{dt}}=D\frac{{d}^{2}\varphi }{{{dx}}^{2}}.$$

The rate of the change in gas concentration at a particular depth ($d\varphi /{dt}$) depends on the diffusion coefficient and the second derivative of the gas concentration with depth, which varies for each gas-polymer combination. In the previous work, we have developed a well fitted model to describe the adsorption and diffusion of molecules in polymer. This model predicts also time-dependent variations in non-static vapor concentration in the testing chamber^20^. In this study, we simply consider molecular diffusion in polymer. The proportional relation of the mass increment of polymer and the electrical response due to swelling of polymer. Then the calculated curves regarding estimated mass increments in time and the response curves of Δ*V*/*V*_0_ obtained by experiments were compared, in order to estimate the diffusion coefficient, $D$, of penetrant in the observed polymer. To calculate the mass increment in the polymer, the mass per depth was calculated with a constant resolution (100 nm) and integrated discretely for the entire polymer film thickness.

#### Principal component analysis

Principal component analysis (PCA) was performed using the build-in PCA application in Origin R2022. The data was collected by exposing the samples to different gases for 30 s. Data points were recorded at 5-second intervals from the start of exposure until the signal recovered to its ground position. This data set was then used for calculating PCA.

## Results and discussion

### Response to humidity, volatile gases, and other gases

The output patterns of the nanomechanical multisensory array, which integrates multiple sensors on a single chip, provide more accurate and reliable results. Therefore, the fabricated sensors were then evaluated to measure their response to various vapors using a gas flow meter^20,26^.

**Fig. 3:**
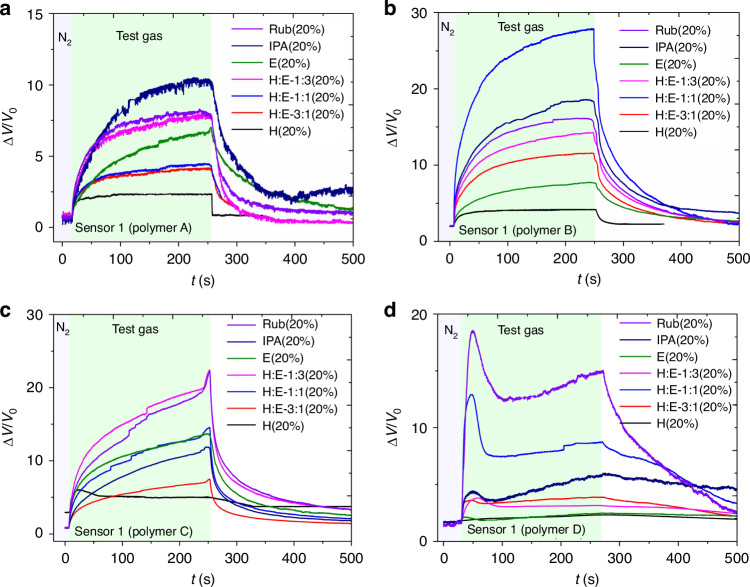
Sensor responses of different polymers to gas exposure. Responses of the Polymer A (**a**), Polymer B (**b**), Polymer C (**c**) and Polymer D (**d**) deposited on different sensors for exposure to 20% of different gases. Here, H, E, IPA and Rub mean humidity, ethanol, isopropanol, and hand rubbing alcohol, respectively

**Fig. 4:**
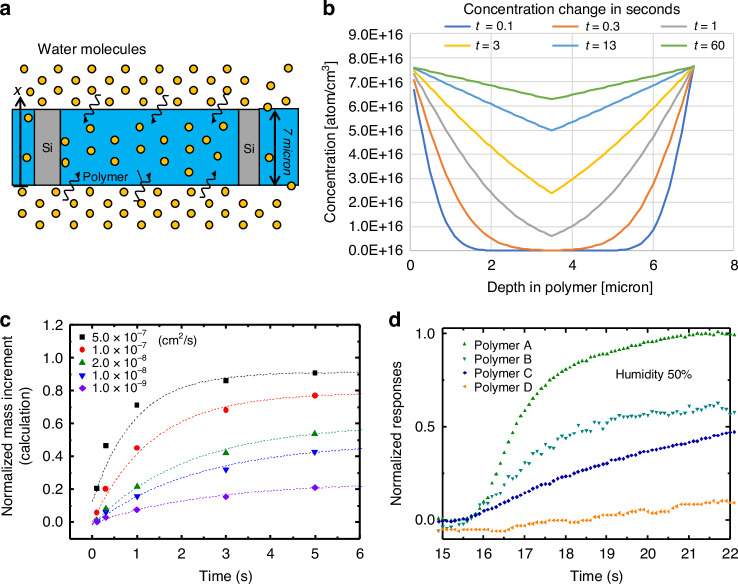
Molecular diffusion and water sorption dynamics in polymer-based sensors. **a** Illustration depicting the diffusion of molecules within the polymer in the sensor slits. **b** Concentration profiles of water molecules, displaying changes in seconds for penetration from both sides. **c** Normalized mass increment based on sorbed water mass shows the time dependence for different diffusion coefficients of water within the polymer. (**d** Magnified graph of normalized experimental responses illustrating the first 7 s after exposure for 50% of humidity test gas

The experiment involved the use of different gases and organic liquids that were vaporized using a bubbler and then passed through a gas chamber where the sensors were subjected to them. The test gases with mixture vapors were prepared by bubbling with H_2_O, ethanol, a mixture of H_2_O:ethanol in the ratios of 1:3, 3:1, and 1:1, IPA, H_2_ gas, CH_4_ gas, and hand rubbing alcohol (water:ethanol:IPA = 18:78:4) in the bottles. The response of each sensor was recorded as Δ*V*/*V*_0_ (the change in voltage output of the sensor relative to the initial voltage value V_0_) *vs* time (s) for each vapor in Fig. 3a–d and plotted in a single graph in Fig. 4a–d, resembling solubility patterns in the sorption model^37^.

The *ΔV*/*V*_0_ value is a measure of the response of the sensor to the specific vapor. Figure 3a–d show the responses of the sensors with Polymer A, Polymer B, Polymer C and Polymer D exposed to the test gas/N2 mixtures (20% by volume of test gas). The responses to 4% and 50% by volume of test gas are shown in Supplementary material 1 Figure. S5. The graphs show that the response of the sensors varied significantly depending on the type of vapor being tested. Focusing on the initial 10 s after exposure to the test gas, sensor 4 (Polymer D) showed the highest response to all types of gases. As a result, *ΔV*/*V*_0_ saturates quickly, followed by another diffusion stage. This is attributed to the specific gas-polymer interaction in Polymer D^38^.

When the concentration of the test gases increases from 4% to 50% by volume, the response of the sensor 4 (Polymer D) increases, which is consistent with Henry’s law^20^. As the gas concentrations increase, the dissolved gas concentration in the polymer increases, which leads to a higher response of the sensors. This concentration effect is a key factor in understanding how the gas sensor array can be used for detecting gases under different environmental conditions^20^. In the case of water:ethanol gas mixtures, synergistic response of Polymer A (Fig. 3a), Polymer B (Fig. 3b) and Polymer C (Fig. 3c) is seen: some or all mixtures produce signals which are stronger than signals of both pure water and pure gaseous ethanol.

It was observed that the response of the sensors to different gases increases for all polymers when exposed to higher concentrations of the gases, which is consistent with Henry’s law. The sensor array showed varying responses with different output voltages to different vapors, indicating that it has the potential to be used for detecting and analyzing various gases and vapors in different environments. A demonstration of sensing water vapor and Ethanol vapor is shown in Supplementary material 3.

### Sensitivity

The sensitivity of the sensors is defined by the volume change of the polymer in response to gas exposure, as described above. Experiments with gas with different concentrations (4%, 10%, 20%, 50%, and 100%) also produce a clear concentration gradient due to their interaction with the polymer. This range of concentration gradients leads to various diffusion rates and fluxes, consequently affecting the sensor’s output change and, thereby, its sensitivity.

The sensors were additionally exposed to both 2% H_2_ and 2% CH_4_ gases, exhibiting robust and effective responses. The Supplementary material 1 Figure. S6 shows the response of the 4 different sensors when exposed to 2% H_2_ and 2% CH_4_ gases. However, diffusion of these molecules into the polymer does not result in a significant sensor response.

The model in Fig.4 a, in which sorption of molecules occurs on both the top and bottom surfaces of the polymers is adopted for calculations. The polymer thickness was 7 microns based on the thickness of the Si layer on the sensors. The concentration, assuming various diffusion coefficients, is estimated across the material’s depth due to diffusion over time. Here, the voltage change, a polymer’s response signal Δ*V*, is assumed proportional to the mass increment resulting from the molecules sorbed within the polymer matrix. The diffusion coefficients were estimated by comparing the increment curves of mass and Δ*V*/*V*_0._ As shown in Fig. 4b, concentration profiles can be obtained by calculating concentrations at various depths from the surfaces over time. It is observed that within the initial one second, water molecules do not reach the center of the polymer body. However, during the subsequent 60 s, water molecules spread throughout the entire polymer body. Analysis of the water profile within the polymer allows estimation of the absorbed water mass, as presented by the normalized mass increment in Fig. 4c. It’s evident from the calculation that saturation tends to occur within less than 10 s for a higher diffusion coefficient exceeding 5.0 × 10^-7 ^cm^2^/sec. In Fig. 4(d), the normalized response curves obtained from Fig. 3 are magnified to highlight the first 7 seconds responses after exposure for 50% of humidity gas. The graph reveals that the Polymer C exhibits a diffusion coefficient within the range of 1.0 × 10^−^^7^ and 5.0 × 10^−^^7 ^cm^2^/sec. This range would be acceptable compared to the experimental value between 1.1 and 1.8 × 10^−^^8^ cm^2^/sec at 30 ^o^C^34,39^. The diffusion coefficient of water in Polymer A varies from 10^−^^7^ to 10^−^^8 ^cm^2^/sec. Whereas Polymer B and Polymer D show even lower diffusion coefficients, likely below 10^−^^9 ^cm^2^/sec, and 10^−^^9 ^cm^2^/sec without clear response intensity, respectively.

The diffusion and swelling of water molecules in the polar polymer, Polymer C, are greater than in other polymers because of it’s moderate interaction with water molecules and its amorphous (non-crystalline) structure. This structure allows water molecules to penetrate easily between the polymer chains, leading to swelling. On the other hand, Polymer A with non-polar hydrocarbon chains have limited interaction with water molecules, while Polymer D can form strong hydrogen bonds with water molecules, which may make it easier for water molecules to stay inside the polymer and less likely to diffuse out. These differences show that the chemical properties and physical structure of polymers have a significant influence on the behavior of water molecules.

**Fig. 5:**
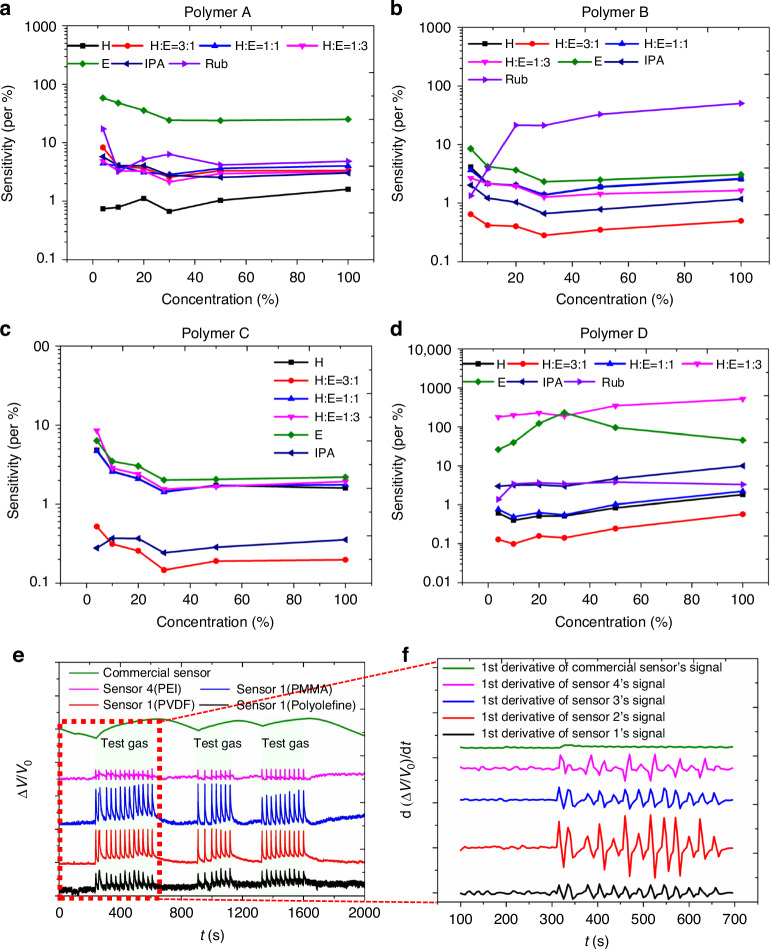
Sensitivity and response dynamics of polymer-based gas sensors under modulated gas flow conditions. Sensitivity of the sensors for the polymers deposited by **a** Polymer A (Polyolefin), **b** Polymer B (Fluorocarbon polymer), **c** Polymer C (Acrylic resin) and **d** Polymer D (Amino polymer) for different gas exposure. **e** The response dynamics of sensors comprising four distinct polymers and a commercial sensor. The plot depicts the phase of turning the gas flow (ethanol) on and off at two-second intervals. **f** The rate of change in voltage d(ΔV/V0)/dt for four polymer-based sensors and a commercial sensor (Organic Solvent Vapor Sensor, model-TGS2620, Figaro Engineering Inc.). The conditions remain identical, with the gas flow modulated at the specified two-second intervals

The sensitivity of the sensor is represented by the following equation^20,36^

3 $${S}_{\varepsilon }=\frac{\frac{\Delta V}{{V}_{0}}}{\varepsilon }$$

where${\rm{where}}$ *ε* represents the linear expansion ratio of the polymer. as shown in Fig. 5. It is evident that there is a significant variation in the sensitivity of each sensor towards different gases and gas mixture. This can be determined from the change in voltage of the sensor due to the expansion of the polymer for different gas-polymer pairs. Firstly, the individual response of the sensors from a single gas species is considered. The sensors utilizing Polymer A (Fig. 5a), Polymer B (Fig. 5b) and Polymer C (Fig. 5c) exhibit a high sensitivity to a wide range of ethanol concentrations. On the other hand, the sensor using Polymer D (Fig. 5d) exhibits a high average sensitivity to a gas mixture of humidity (water vapor) and ethanol in a 1:3 ratio. In terms of humidity (water vapor), the overall sensitivities are reduced compared to ethanol. Specifically, the sensitivity of Polymer A and Polymer D is significantly reduced compared to Polymer B and Polymer C, which exhibit a lesser reduction in sensitivity.

Ethanol molecules have a polar hydroxyl group and a non-polar hydrocarbon chain, making them amphiphilic. This characteristic allows ethanol to dissolve or diffuse easily in both polar and non-polar environments^32,40^. The maximum of the signal is expected to be proportional to the equilibrium solubility when diffusion is fast on time scale of experiment. Therefore, ethanol can penetrate polymers with varying polarities, such as the polar Polymer C and Polymer D, and the non-polar Polymer A and Polymer B, by interacting with different functional groups and fitting into the polymer matrix due to its moderate size and polarity. Ethanol molecules may interact strongly with Polymer A due to its medium polarity and small molecular size.

Water molecules are highly polar and can form strong hydrogen bonds with each other. This high polarity and strong hydrogen bonding make water less compatible with non-polar polymers like Polymer A and Polymer B, as these polymers lack the functional groups necessary to interact effectively with water. While Polymer C and Polymer D are more polar and can interact with water somewhat, the strong self-association between water molecules via hydrogen bonding still limits their diffusion compared to ethanol. Polymer A interact weakly with polar molecules, especially with highly polar molecules like water. Water molecules are, therefore, less likely to penetrate between Polymer A chains and cause swelling.

Due to its lower polarity or lager molecular size, IPA may not swell highly polar polymers as effectively as methanol or water, however it can penetrate specific polymers more efficiently. Since the diffusion coefficient also depends on molecular size and shape, further experimental analysis is required to consider the appropriate response for the adsorbed molecules and polymer matrix.

The individual response of the sensors to a mixture of gas species is considered. Sensors integrated with Polymer A, Polymer B, and Polymer D exhibit different sensitivities to concentrations of rubbing alcohol and (H:E) of 1:3. The sensor with Polymer A shows no significant difference in response to these mixture vapours. However, the sensor with Polymer B shows a higher sensitivity to rubbing alcohol compared to the (H:E) of 1:3. On the other hand, the sensor with Polymer D exhibits higher sensitivity to the (H:E) of 1:3 compared to rubbing alcohol. Diffusion of multiple molecules may exhibit different swelling properties than for single molecules. For example, water is very polar, and ethanol is moderately polar. When polymers are partially polar, a mixture of water and ethanol can optimize their interaction with polymers and more effectively penetrate between polymer chains to cause swelling than molecules on their own^41^. Such behavior depends on the type of molecule and the mixing ratio of the molecules.

The differences in sensitivity among the various sensors under different gas vapor concentrations reinforce the impact of gas-specific and polymer-specific diffusion properties on the sensor performance. It illustrates the practical implementation of theoretical principles, enabling potential applications where high sensitivity and accuracy are essential.

For a more detailed and nuanced understanding, additional studies could delve into the specific diffusion coefficients of the gas-polymer pairs and how they impact the observed results^20,42–46^.

### Response time and recovery time

The response time and recovery time of the four sensors made of different polymers were evaluated for different gas vapor concentrations of the organic liquids tested, including H_2_O, ethanol, a mixture of H_2_O: ethanol, IPA, and hand rubbing alcohol.

The response/recovery times are related to the polymer-gas molecules interaction, which can generally be considered as being influenced by the diffusion of gas molecules into and out of the polymer matrix, which has been modeled by Fick’s second law of diffusion in Eq. (2).

The response time (*t*_s_) and recovery time (*t*_r_) can be roughly estimated as the time it takes for the gas to diffuse into and out of the polymer, respectively, which can be estimated^20,40^,

4 $$t\approx ({L}^{2}/6D),$$

where *L* is the characteristic length (e.g., the thickness of the polymer layer and *D* is the diffusion coefficient.

The response time of each sensor is determined by measuring the time taken for the sensor to reach 90% of its maximum sensitivity after exposure to the gas vapor in Fig. 3. Similarly, the recovery time was determined by measuring the time taken for the sensor to return to its baseline sensitivity level after the gas vapor was removed. The sensors with Polymer A, Polymer B, Polymer C, and Polymer D exhibit an impressive swift response time to various gas exposures. Specifically, response times of 38 s were recorded at 4% ethanol concentration, 53 s at both 4% and 20% humidity levels, 6 s at a 50% humidity level, and 5 s at 50% ethanol concentration. Moreover, the recovery times of these sensors serve as an indicator of their effectiveness and efficiency, showcasing their ability to return to operational status fast and reliably. Specifically, Polymer A achieves a recovery time of 3 s at 20% humidity, Polymer B requires 41 s at 20% humidity, Polymer C takes 78 s at the same humidity level, and finally, Polymer D necessitates 66 s at recovery with Isopropyl Alcohol (IPA) which are much faster than the reported gas sensors which are^47,48^.

The response of the sensor when the gas is switched on and off can be used as important information to recognize the gas type, which is also included in the subsequent PCA analysis.

Comparing to similar reported literature, the sensitivity, response time, and recovery time of this work’s sensor are superior shown in Table 1. Specifically, the Si-polymer nanomechanical sensor in this study exhibits a wide sensitivity range (90–500%), a quick response time (3 s), and a fast recovery time (5 s) at room temperature (26 °C), outperforming other materials in terms of efficiency and adaptability for multiple gases comparing to the reported literatures^12,19,20,47–50^.

**Table 1:** Comparison of sensitivity, response time, and recovery time with similar reported literature

Materials	Sensing Mechanism	Sensitivity (%)	Operating Temp (^o^C)	Response time (s)	Recovery time (s)	Target gas	Refs
Si-Polymers	Nanomechanical Stress on Piezoresistive part by absorbing gas	90–500	26	3	5	Multiple	This work
BaTiO3–CuO thin-film	Change of impedance of Metal-semiconductor junction absorbing gas	22-24	300	–	–	CO2	^60^
Si-Polymer	Surface stress by absorbing gas	2.5	18	162	–	Humidity	^19^
Si-Polymer	Surface stress by absorbing gas	2.2		440	1200	Organic gases	^20^
	Nanomechanical Membrane-Type Surface Stress	40	19	25	30	Smell of spices	^12,61^
N-doped graphene quantum dots and polymer composite	N-doped graphene quantum dots and polymer composite	120		164, 12	40, 32	Methanol	^62^
MOF-derived carbon NPs decorated mesoporous a-Fe2O3 NRs	redox reactions, with reducing gases releasing electrons, lowering resistance.	5.2	20	10	27	Multiple	^63^

### Repeatability

In order to test the repeatability of the nanomechanical multisensory arrays, an experiment was conducted where after being exposed to gases for 2 s, the sensors were subjected to a cessation of gas flow until the voltage signal reverted to its baseline. The sensor exposure to gas and subsequent cessation of gas flow was iterated for 470 s, followed by an interruption of 282 s. This entire sequence was replicated, completing two analogous cycles, and the sensors’ responses were recorded and graphed as Δ*V*/*V*_0_ vs. time for each sensor.

The experiment was conducted for all four sensors in the gas sensor array, along with a commercial sensor for comparison. The data obtained for each sensor was plotted in Fig. 5e, representing the response of a particular sensor (polymer) to repeated gas exposure and turn-off cycles.

From Fig. 5e, it was observed that the response of the sensors remained consistent throughout the repeated cycles of gas exposure and turn-off. The Δ*V*/*V*_0_ values for each sensor remained within a relatively narrow range throughout the experiment, indicating that the sensors were highly repeatable in their response to gas exposure. Conversely, responses of the commercial gas sensors under repeated gas exposure are also shown in Fig. 5e, f)^49,50^.

Furthermore, the dv/dt values were calculated and plotted vs time, as shown in Fig. 5f for all four sensors and the commercial sensor. The dv/dt value represents the rate of change in the voltage output of the sensor, and it is a measure of the sensitivity of the sensor to gas exposure. From Fig. 5f, it is observed that the dv/dt values for each sensor were consistent throughout the repeated cycles of gas exposure and turn-off, indicating that the sensors were highly sensitive and repeatable in their response to gas exposure^51,52^.

### Experiment with fish

In this experiment, nanomechanical multisensory arrays were used to monitor the quality of mackerel fillets over a seven-day period in a way similar to ref.^53^. The experiment began on day one, immediately after cutting and storing the mackerel fillets, with subsequent measurements taken on the third, fifth, and seventh days of storage. The gas flow system was crucial for measuring the gases released from the mackerel fillets. The chopped fish fillets were stored in a glass vessel equipped with two ports to facilitate effective airflow control. Nitrogen gas was introduced into the vessel through the inlet port, carrying the gases emitted from the fish. These gases, mixed with nitrogen, then flowed through the outlet port and into the test chamber, where the gas sensor arrays detected and analyzed the emitted volatiles. The entire experiment was conducted at room temperature, and the detailed experimental setup is depicted in Supplementary Material 1, Fig. S7.

**Fig. 6:**
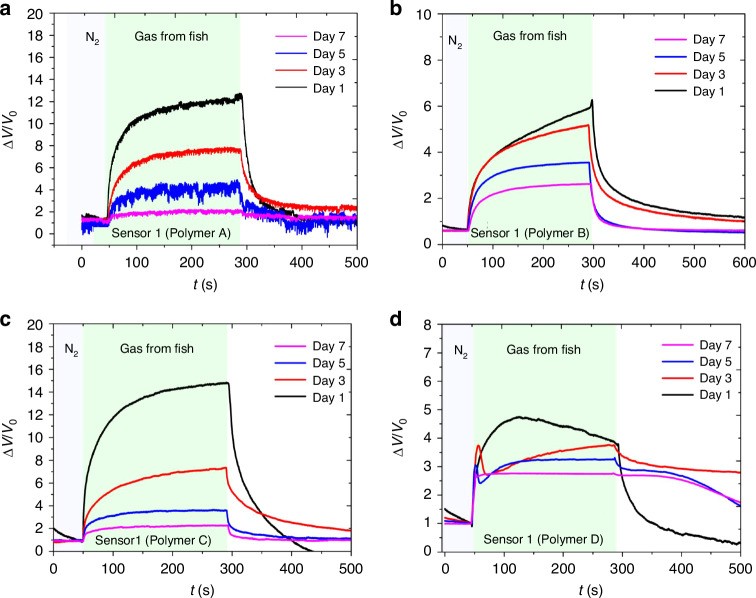
Sensor responses to fish emitted gases over storage period (days 1–7). Relative voltage change ratio, represented as ΔV/V_0_, of the four disparate sensors (**a** sensor 1, **b** sensor 2, **c** sensor 3 and **d** sensor 4) exposed to gases emitted from fish during a storage period spanning from day one to day seven. This graphically elucidates the sensor responses to the evolving biochemical conditions of the fish over this specific timeframe

The response of each sensor was recorded and plotted as Δ*V*/*V*_0_ vs time for each day in Fig. 6. From the graphs, it was observed that the intensity of the Δ*V*/*V*_0_ values for each sensor decreased from day 1 to day 7. This decrease in intensity was most significant for the Polymer D and Polymer C sensors. The microbial activity generated during fish spoilage is known to produce a variety of inorganic gases and volatile organic compounds^54^. A very large number of gases are involved in the response of the sensor, and the sensor response is the combined effect of these gases.

**Fig. 7:**
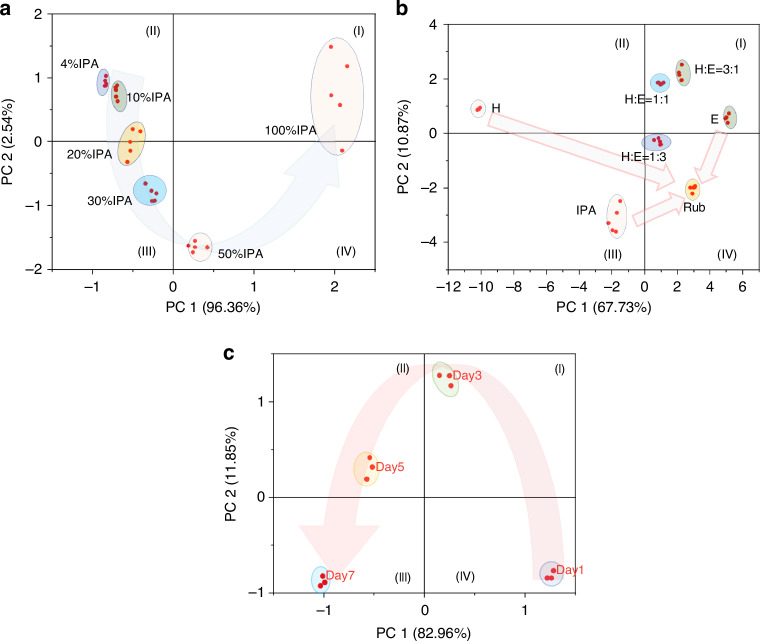
Principal component analysis (PCA) for distinguishing gas concentrations and individual gas components from complex gas mixtures. The PCA plot depicts two distinct analytical scenarios: **a** PCA differentiation based on varying gas concentrations, and **b** PCA separation of single gas components within gas mixtures. **c** The PCA of gases released from mackerel fish over 7 days period. Gas mixtures analyzed include humidity (H), ethanol (E), isopropyl alcohol (IPA), and rubbing alcohol (Rub)

Amine gases are one of the gases typically released as fish begins to deteriorate and play a significant role in the overall gas mixture detected by the sensor array. The analyzed plots reveal a distinct directional and curvilinear response for these volatile components, as highlighted in Fig. 7c. This behavior indicates that the sensor array effectively captures the complex dynamics of gas composition changes during spoilage, including contributions from amines, without the need to isolate individual gas components. This capability allows for real-time monitoring of spoilage processes, which is critical for practical applications such as optimizing storage conditions or detecting spoilage early.

By focusing on the combined gas mixture rather than individual gas identification, the sensor array demonstrates its robustness in detecting spoilage under real-world conditions. This dynamic detection approach ensures effective monitoring of the gas composition over time, making it possible to assess spoilage progression accurately and efficiently. Such versatility makes this system highly suitable for applications where rapid and reliable quality control is essential, such as in food storage and transportation.

The results of this experiment suggest that the gas sensor arrays can be used to monitor the quality of the fillets over time. As the fish deteriorates, different gases are released from it, which can be detected and measured by the gas sensor arrays. The decrease in the intensity of the Δ*V*/*V*_0_ values for each sensor over time indicates that the gas composition of the fish changes as it deteriorates^55^.

By monitoring the changes in the gas composition of the fish using the gas sensor arrays, it may be possible to determine the optimal storage conditions for the fish to preserve its quality for longer e.g. in cold transport. Moreover, the gas sensor array could potentially detect spoilage in the fish even before it becomes visibly noticeable or identifiable by smell. This approach appears more streamlined than other reported systems, primarily because our methodology utilizes a single sensor array to detect many gases. In contrast, many studies reported thus far have employed multiple bulky sensors and complex systems, making our approach notably more efficient^54,56^.

### Principal component analysis (PCA) analysis

Principal Component Analysis (PCA) is a statistical method used to analyze large data sets by reducing the data to its principal components. In the context of gas sensor arrays, PCA can be used to identify patterns in the data that relate to different gas concentrations, allowing for more accurate and efficient detection of different gases^57^.

In this experiment, PCA was calculated for all individual test gases described above in different concentrations where gas mixtures are considered as individual test gases^57^. The data obtained from the gas sensor arrays was first preprocessed to remove any noise or inconsistencies in the data. The resulting data was then analyzed using PCA to identify the principal components that explain the variance in the data.

From the PCA analysis shown in Fig. 7a, it was observed that the different gas concentrations of IPA could be differentiated based on their respective principal components. Observations show that 4% of IPA dispersed into the center of the I quadrant, with an additional 10% IPA located beneath this 4% in the same quadrant. There is a scattering of 20% IPA across both the I and II quadrants. Furthermore, 30% and 40% of IPA can be seen dispersed into the II and III quadrants, respectively. The majority of the 100% IPA predominantly dispersed in the IV quadrant. The concentrations of different gases were found to have unique patterns of principal components, which could be used to accurately detect and differentiate between different gas concentrations, similar to observations made in studies by Jones et al.^33,57^.

Figure 7b illustrates the PCA analysis of all gases tested at identical concentrations and under similar conditions. It is seen that H and IPA scattered into the I and II quadrants, H:E = 1:3 and rubbing alcohol (W/E/I = 18:78:4) are seen to be dispersed in III quadrants but in different areas. H:E = 1:1 scattered in IV quadrants but near I quadrant while H:E = 3:1 scattered in middle of the IV quadrant and E scattered in the IV quadrant closer to the II quadrant. The analytes exhibit distinct patterns across the PCA plot, supporting the premise that different gases or mixtures produce unique scattering patterns, which can be used for identification^34^.

Figure 7c illustrates the Principal Component Analysis (PCA) of gases emanating from mackerel fish, monitored over a 7-day period at room temperature, from fresh to spoiled stages. Initially, data from the fresh mackerel clusters in the lower right of quadrant III, which then migrate upward after 3 days before descending, with spoiled fish data clustering at the bottom of quadrant II. These trends align with previous studies, validating the effectiveness of our sensor arrays in capturing these gases, thereby providing an efficient method for tracking fish freshness^58,59^.

The PCA analysis also showed that the gas sensor arrays could be used to detect and differentiate between different gas concentrations with high accuracy and efficiency. Furthermore, by creating an algorithm based on PCA analysis, the gas sensor arrays could be automated to detect and analyze gas concentrations in real-time, without the need for human intervention. The sensors demonstrate strong reproducibility in repeated measurements, as shown in Supplementary Material 1, Figure. S8. The standard reproducibility factors are Polymer A: 0.0072, Polymer B: 0.0243, Polymer C: 0.0140, and Polymer D: 0.0143 which implies the sensor’s high repeatability.

## Conclusions

This study illustrates the promise of microfabricated nanomechanical multisensory array, in the realm of multiple gas detection. By employing silicon and polymer-based sensors within an SOI wafer, a multi-target gas sensor arrays are developed. Four different types of polymers - Polymer A (Polyolefin), Polymer B (Fluorocarbon polymer), Polymer C (Acrylic resin), and Polymer D (Amino polymer) are deposited on the sensor array’s silicon slits, thereby demonstrating the sensor’s potential and accuracy for diverse gas detection. The nonlinear responses suggest the potential for performance optimization through advanced ML techniques, enhancing their applicability in diverse gas detection scenarios. The four sensors demonstrate different sensitivity and response time across a range of gas vapor concentrations, highlighting their efficacy in real-time gas detection. The gas sensor arrays maintain a consistent and repeatable response throughout repeated gas exposure and shutdown cycles, indicating their high reliability. Through principal component analysis, unique gas sensor array data patterns allowed precise identification and differentiation of various gas concentrations, suggesting the potential for creating an autonomous real-time gas or vapor detection system. The sensor array has potential for use in various fields, such as the food sector, healthcare, environmental monitoring, and anomaly detection in factories, etc.

## Supplementary information

Supplimentary Material 1

Supplementary material 2

Supplementary material 3

## Data Availability

The data that support the findings of this study are available in this manuscript and supplementary material of this article.
