# Supplementary material for: Investigation towards nanomechanical sensor array for real-time detection of complex gases
Source: Microsyst Nanoeng. 2025 Mar 24;11:53. doi: 10.1038/s41378-025-00899-2 (PMC11930958; doi:10.1038/s41378-025-00899-2)
Supplement: Supplementary file 1 — Supplimentary Material 1 [file 41378_2025_899_MOESM1_ESM.docx]

**Investigation Towards Nanomechanical Sensor Array for Real-time Detection of Complex Gases**

Md. Abdul Momin^1,2,a^, Masaya Toda^1b^, Zhuqing Wang^1^, Mai Yamazaki^3^, Krzysztof Moorthi^3^, Yasuaki Kawaguchi^3^ Takahito Ono^1c^

^1^Graduate School of Engineering, Tohoku University, 6-6-01 Aramaki-Aza-Aoba, Aoba-ku, Sendai, 980-8579, Japan.

^2^Depatment of Bioengineering, University of Pittsburgh, 4200 Fifth Ave, Pittsburgh, PA 15260, USA

^3^R&D Center, Mitsui Chemicals, Inc., 580-32 Nagaura, Sodegaura, Chiba, 299-0265, Japan.

Corresponding authors:

^a^[mdm201@pitt.edu](about:blank) (Momin, M. A.), ^b^[toda@tohoku.ac.jp](mailto:toda@tohoku.ac.jp) (Toda, M), ^c^takahito.ono.d4@tohoku.ac.jp (Ono, T)


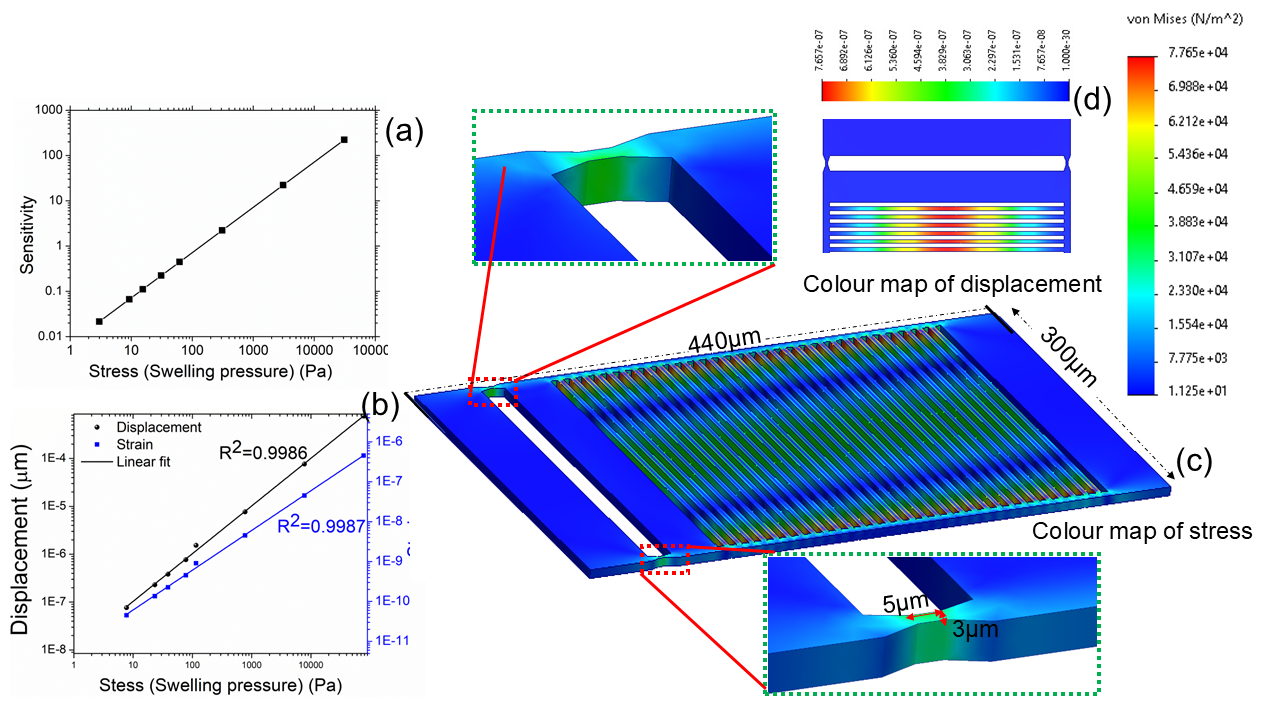


Fig. S1 (a) Computed structural sensitivity of the piezoresistive sensor component, revealing a proportional increase with the applied swelling pressure (stress) induced by the polymer's absorption of gas molecules, (b) Modeled stress-strain and stress-displacement curves of the sensor, both demonstrating linearity in response to swelling pressure (stress), (c) Color map illustrating simulated stress distribution across the piezoresistive components and Si slits resulting from the polymer's applied swelling force. The maximum stress is concentrated on the beams of piezoresistive parts, enhancing sensor sensitivity, (d) Simulated color map depicting the corresponding generated displacement of the sensor, revealing that the maximum stress occurs in the silicon slits due to the polymer's swelling force.


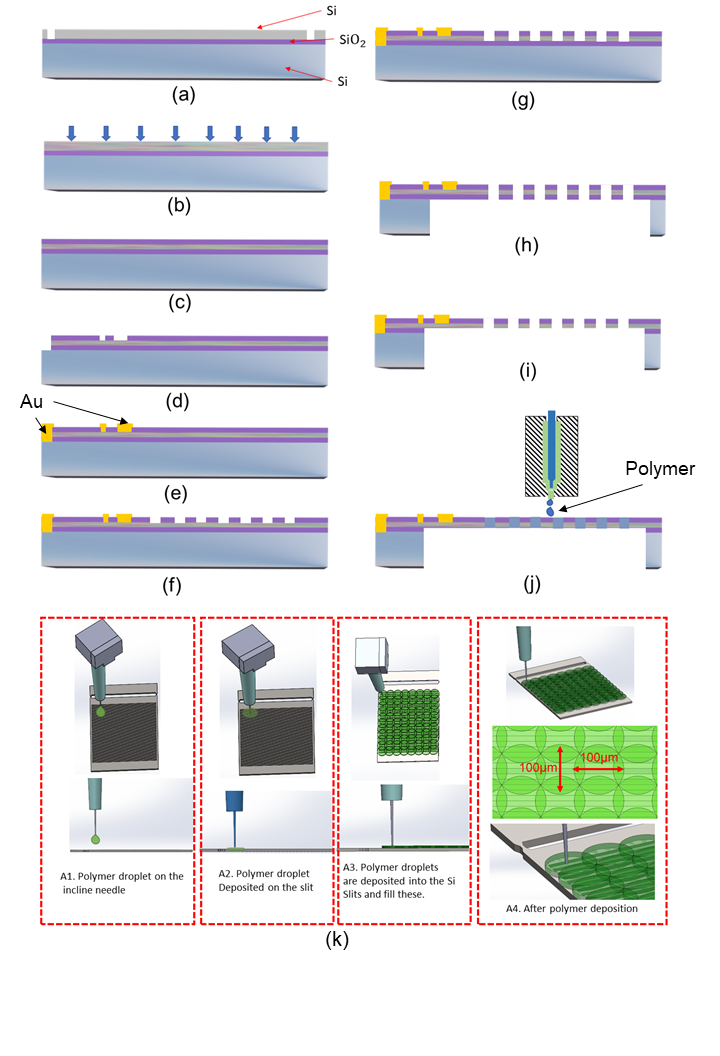


Fig. S2 Fabrication process of the gas sensor array. (a) Mark alignment by etching Si, (b) ion implantation, (c) SiO_2_ deposition using CVD, (d) SiO_2_ etching using BHF for opening windows for electrodes, (e) Cr/Au deposition using sputtering and then etching using wet etching, (f) SiO_2_ etching for making slits, (g) device layer Si etching for making slits, (h) back side Si etching, (i) backside SiO_2_ etching, (j) finally polymer deposition. (k) the polymer deposition method using a microscopic coating applicator instrument.

1. **Fabrication of the chip**

The fabrication process of nanomechanical gas sensors (supplementary Fig. S2 (a-j) has been done on an SOI substrate [16, 23] made of Si/SiO_2_/Si (7/1/400 µm in thicknesses). Firstly, the device layer is implanted with boron ion (using a BF_6_ gas source) at acceleration voltages of 80 and 40 keV at a dose of 1×10^14^ and 3×10^14^ cm^-2^, respectively. It is then annealed at 850 and 1050 ^o^C for 30 and 3 min, respectively. Then 300 nm-thick SiO_2_ is deposited using a tetra ethoxy silane chemical vapor deposition, and the SiO_2_ layer is patterned to make contact windows for electrode deposition. Magnetron sputtering has been used to deposit 20 nm-thick Cr and 150 nm-thick Au films for electrodes, and unwanted areas are etched using wet-etching. For making the piezoresistive part and the Si slits, a SiO_2_ pattern has been formed by etching SiO_2_ using a buffered HF solution, and then the device layer Si has been etched using inductively coupled plasma reactive ion etching using SiO_2_ as a mask. The backside (handle layer) of the wafer is etched using inductively coupled plasma reactive ion etching. Then SiO_2_ of the BOX layer has been etched using reactive ion etching.

1. **Effect of polymer deposition on the resistance of the sensors**

The resistances of the sensors were initially measured prior to depositing the polymer solution on the Si slits. The resulting resistances were recorded in Table 1, illustrating the values before polymer deposition, after deposition, and following subsequent heat treatments. Notably, the resistances of the piezoresistive components generally increased after polymer deposition. This observed increase is attributed to the slight deformation of the Si slits caused by the deposition of most polymers, resulting in elevated resistance.

To address non-uniform polymer deposition, a two-step deposition process was employed, and additional droplets were strategically utilized to fill any gaps. Manual deposition, while effective, sometimes leads to uneven distribution. Consequently, the Si slits exhibited slight deformation after polymer deposition, further contributing to increased resistance.

However, following a heat treatment period lasting 2 minutes at a maximum temperature of 180°C, the resistance decreased. This reduction is attributed to the restoration of the silicon slits to their initial parallel position, aligning with their pre-deposition state.

For a visual understanding, refer to Fig. S3, showcasing optical microscopic and SEM images before and after heat treatments. These images vividly depict how the polymers fill the Si slits based on their inherent properties.

Table 1 summarizes the measured resistances of the sensors both before and after polymer deposition. Additionally, resistances were measured after subjecting the sensors to heat treatments, providing a comprehensive overview of the sensor performance under different conditions.

| 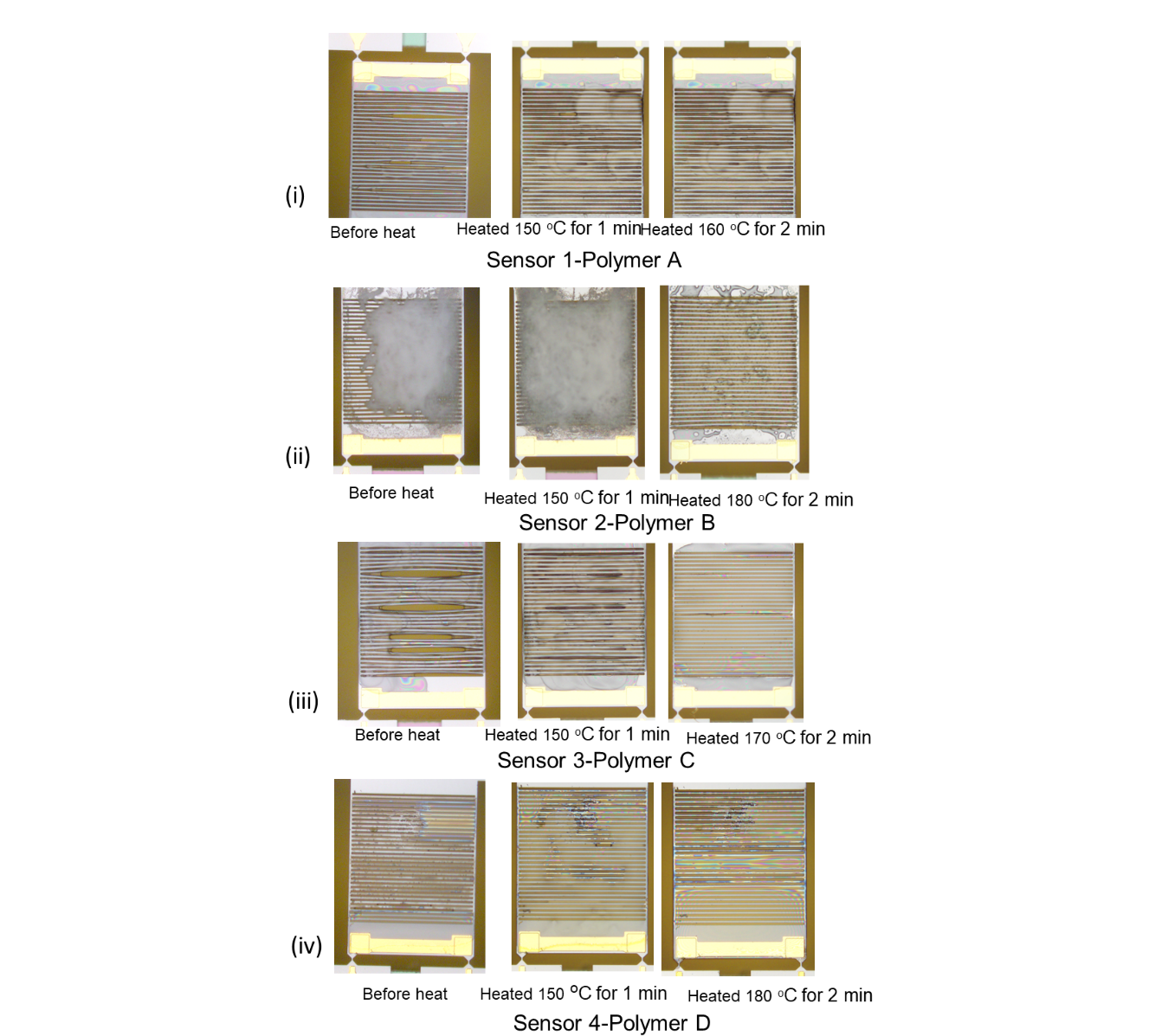 |
| --- |

Fig. S3 Microscopic images of the silicon slits after the polymer deposition and heat treatments where (i) Polymer A (Polyolefin), (ii) Polymer B (fluorocarbon polymer), (iii) Polymer C (Acrylic resin), and (iv) Polymer D (Amino polymer).

Table S1 Measured resistances of the sensors before and after polymer deposition. The resistances are also measured after heat treatment of the sensors.

| Sensor/polymers | R (kΩ) Before  polymer  Deposition | R (kΩ) After  polymer  deposition | R (kΩ) After heated at  160 oC for 1 min |
| --- | --- | --- | --- |
| Sensor1/Polymer A | 5.55 | 5.90 | 5.79 |
| Sensor2/Polymer B | 5.16 | 5.64 | 5.70 |
| Sensor3/Polymer C | 4.28 | 4.74 | 4.57 |
| Sensor5/Polymer D | 4.43 | 4.45 | 4.46 |


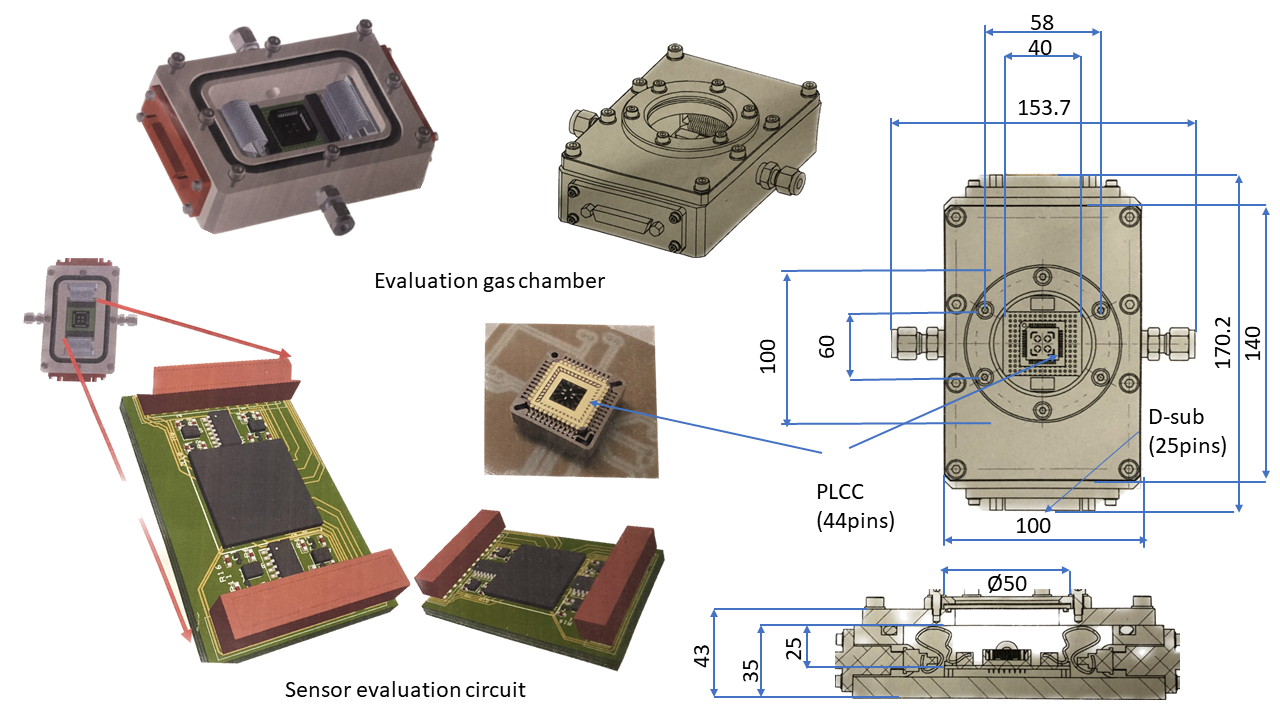


Fig. S4 Size and shape of the evaluation gas chamber and sensor evaluation circuits.


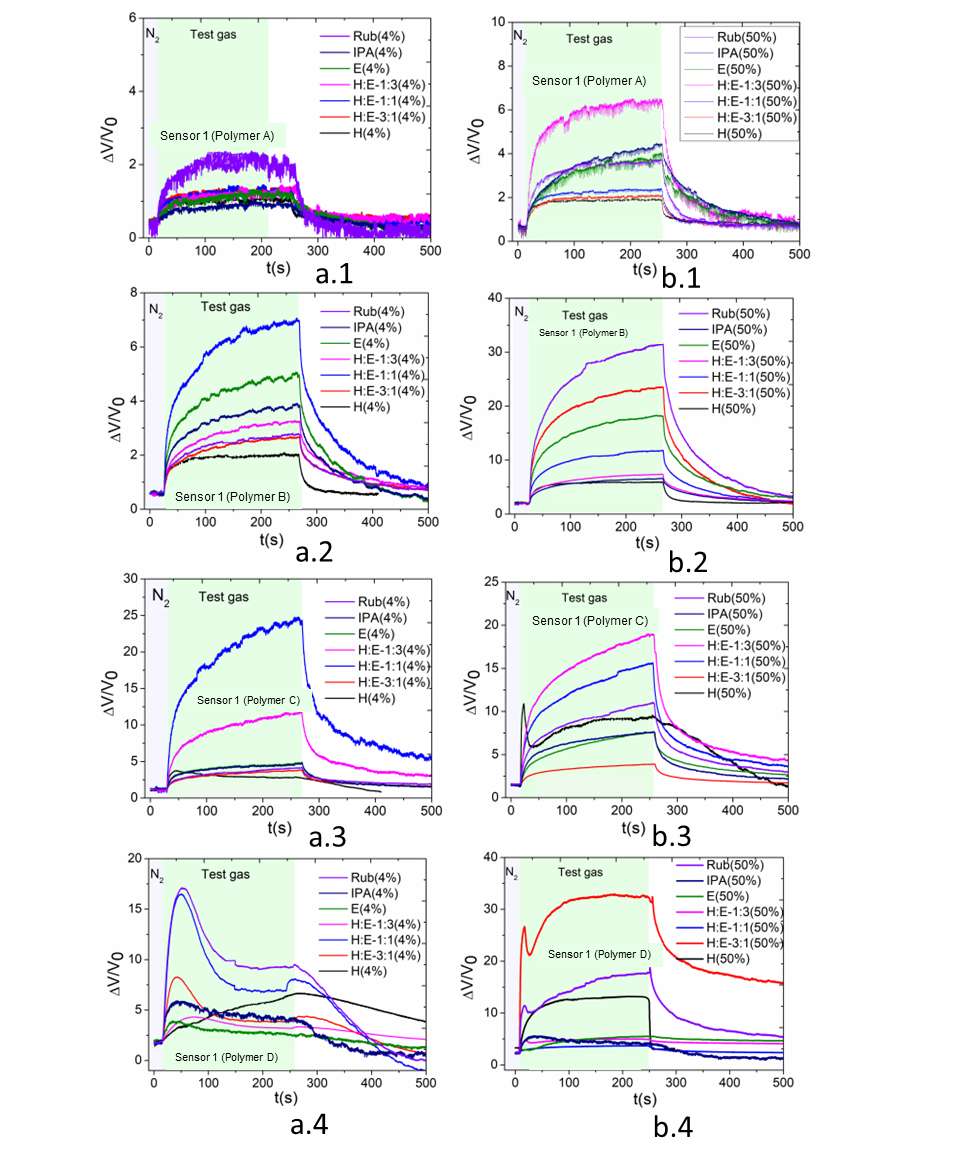


Fig. S5 Fig. 4a(1-4) and 4b(a-4)) are responses of the Polymer A, Polymer B, Polymer C and Polymer C deposited on different sensors for exposure of 4%, and 50% of different gases.


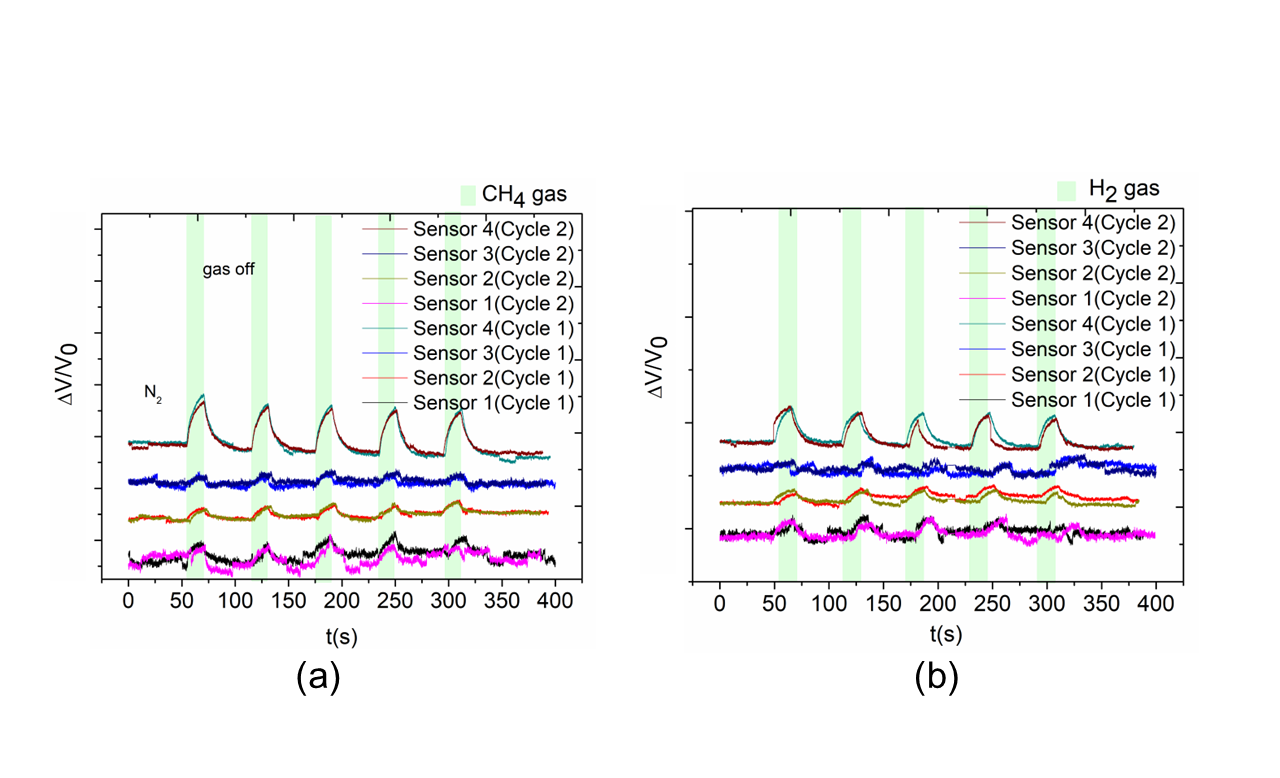


Fig. S6 Illustration of the sensor arrays' performance under the influence of CH_4_ and H_2_ gases. The figure is crafted to visually articulate how the sensors react when exposed to these specific gases, offering clear insights into their characteristic response patterns. The experiment was conscientiously conducted twice to confirm the consistency and robustness of the results. The successful replication underscored the impressive reproducibility of the sensor arrays, thereby strengthening their credibility in accurately detecting and differentiating between CH_4_ and H_2_ gases. This consistency, evidenced by the close alignment of results across both iterations, accentuates the sensors' potential for reliable application in real-world environments where accurate and consistent gas detection is paramount.


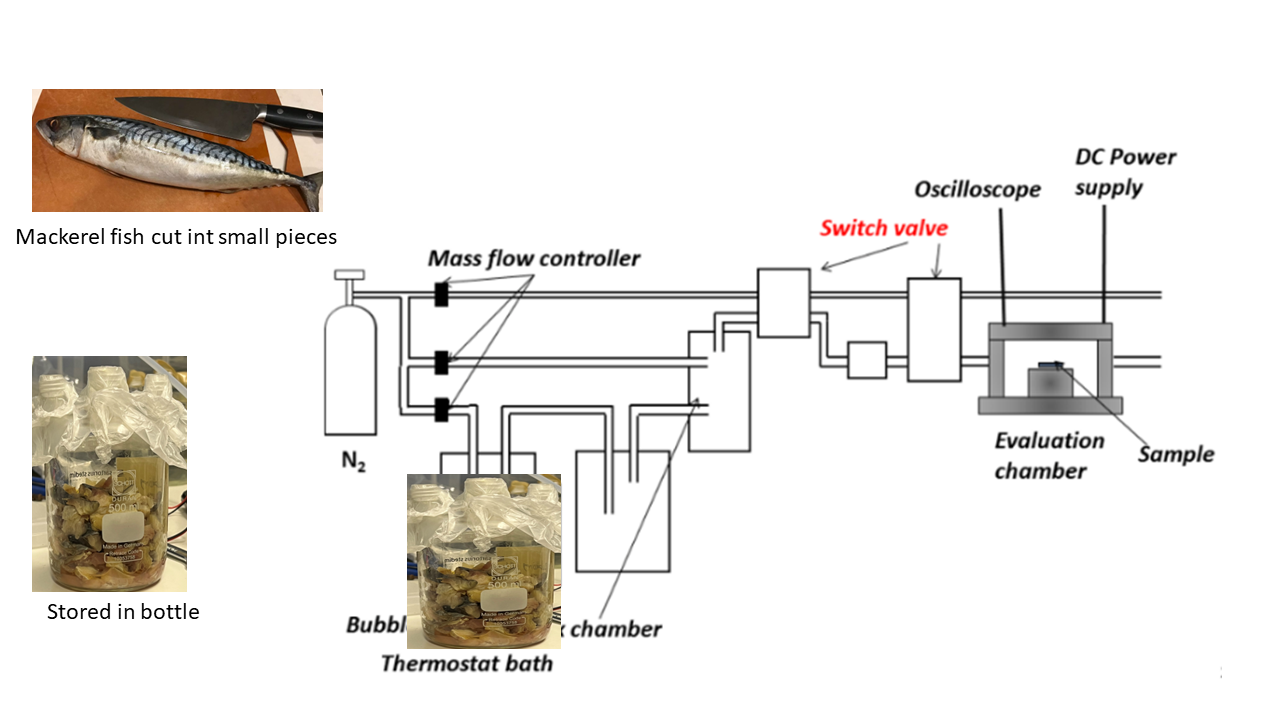


**Fig. S7** Meticulously designed experimental configuration of the gas flow system, developed to evaluate the emission profile of gases released from mackerel fillets over a seven-day period. This rigorous setup combines precise measurement tools and airflow controls, ensuring accurate and comprehensive analysis of gas emission characteristics. The system includes a glass vessel for storing chopped mackerel fillets, fitted with two ports for effective nitrogen flow control. Nitrogen gas is introduced through the inlet to carry the emitted gases from the fish to the test chamber, where the nanomechanical gas sensor arrays perform real-time, multi-gas detection. This configuration demonstrates the practical application of our sensor array for monitoring food quality and spoilage, highlighting its potential for early detection of spoilage gases and optimization of food storage conditions, particularly in cold transport and storage environments.

1. **Reproducibility of the response of the sensors**

The reproducibility of the gas sensors was meticulously confirmed through repeated trials where the sensors were exposed to the various gases five times, with the results demonstrating consistent responses across all exposures, as illustrated in Fig. S8. This consistent response in multiple trials underscores the reliability of these sensors in the accurate detection of a variety of gases.

The sensor array used in this study demonstrates good reproducibility than the other reported research. Sensor reproducibility is maintained in repeated tests [1]. However, the robustness and stability demonstrated by the sensor array in this study highlights its potential as a solution to the reproducibility challenges faced by existing sensor technologies [2].

**3. 1 Standard Repeatability Factor**

The **Standard Repeatability Factor (Overall Average SD) of Polymer A** is calculated as the mean of all standard deviations across all gases and all time points. This metric gives an overall measure of repeatability across the entire dataset of Polymer A.

Here’s the formula step-by-step:

1. **Calculate Standard Deviation for Each Mixture at Each Time Point**: For each gas, the standard deviation at each time point *t* is calculated using:


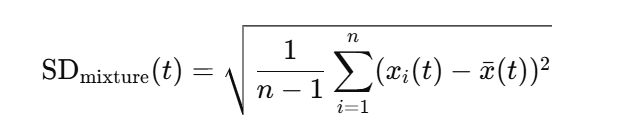


where:

- - *n* is the number of cycles (e.g., 5),
  - $x$_i_(t) is the value for cycle *i* at time *t*
  - $\bar{x}$(t) is the mean of cycles at time *t* for the mixture.

1. **Compute the Average SD Across All Time Points for Each Mixture**: For each mixture, compute the mean of the standard deviations across all time points:


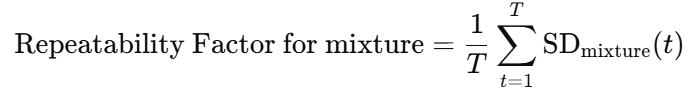


where *T* is the total number of time points.

1. **Combine Standard Deviations for All Mixtures**: Collect all standard deviation values across all mixtures and all time points.
2. **Calculate the Standard Repeatability Factor (Overall Average SD)**: The **Standard Repeatability Factor** is the mean of all standard deviations across all mixtures and time points:


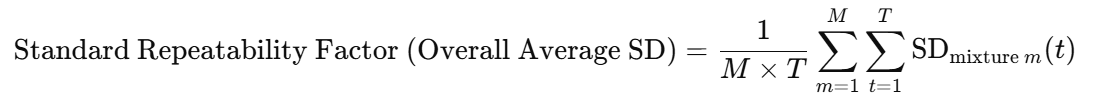


where:

- - M is the number of mixtures,
  - T is the number of time points for each mixture,
  - SD_mixture m_(t) is the standard deviation at time *t* for mixture *m*.

Fig. S8 depicts the consistent performance and reliability of the sensors fabricated using (a) Polymer A, (b) Polymer B, (c) Polymer C (d) Polymer C. The robust reproducibility of these sensors is demonstrated through five successive experimental iterations, solidifying their potential for reliable and efficient gas sensing applications. Here, H, E, IPA and Rub mean humidity, ethanol, isopropanol, and hand rubbing alcohol, respectively.

**Analysis of Repeatability Factors for Each Polymer**

For the four polymers, the repeatability factors are as follows:

- **Polymer A:** 0.0072
- **Polymer B:** 0.0243
- **Polymer C:** 0.0140
- **Polymer D:** 0.0143

These values suggest that the sensors exhibit relatively low variability in repeated measurements, indicating strong consistency. Typically, standard repeatability factors below 0.05 are considered excellent in many sensor applications, as they imply that the sensor can produce stable and precise readings across repeated measurements. Given this, all four polymers demonstrate good repeatability with Polymer 1 showing the highest repeatability among them.

The low standard repeatability factors (especially for Polymer 1) indicate that the sensors based on these polymers can reliably reproduce measurements with minimal drift or fluctuation. This characteristic is crucial in many applications where consistent and accurate readings are essential.

In comparison to other sensors that might have higher repeatability factors (e.g., above 0.05 or even 0.1), these sensors offer superior performance by demonstrating minimal random error. Other sensors with higher repeatability factors might struggle with consistency, showing a broader range of readings even under the same conditions, which could limit their accuracy and dependability.

The repeatability factors achieved here indicate high-quality sensors. Polymer 1’s exceptionally low factor (0.0072) highlights its potential as the most stable and precise choice among the polymers. This low repeatability factor reflects well on the design and quality of these sensors, suggesting they would outperform less consistent alternatives in applications demanding high repeatability and accuracy.

References

1. Liu B, Zhou Y, Fu H, Fu P, Feng L. Lightweight Self-Detection and Self-Calibration Strategy for MEMS Gas Sensor Arrays. Sensors. 2022; 22(12):4315. https://doi.org/10.3390/s22124315.
2. Wang, C., Yin, L., Zhang, L., Xiang, D., Gao, R. (2020). Metal oxide gas sensors: sensitivity and influencing factors. Sensors (Basel), 10(3), 2088-2106.
